# Perfect metamaterial absorber with high fractional bandwidth for solar energy harvesting

**DOI:** 10.1371/journal.pone.0207314

**Published:** 2018-11-12

**Authors:** Mohammad Jakir Hossain, Mohammad Rashed Iqbal Faruque, Mohammad Tariqul Islam

**Affiliations:** 1 Space Science Center (ANGKASA), Institute of Climate Change (IPI), Universiti Kebangsaan Malaysia, UKM, Bangi, Selangor, Malaysia; 2 Centre of Advanced Electronic & Communication Engineering, Universiti Kebangsaan Malaysia, UKM, Bangi, Selangor, Malaysia; University of Science and Technology Beijing, CHINA

## Abstract

A new perfect metamaterial absorber (PMA) with high fractional bandwidth (FBW) is examined and verified for solar energy harvesting. Solar cells based on perfect metamaterial give a chance to increase the efficiency of the system by intensifying the solar electromagnetic wave that incident on the device. The designed structure is mostly offered in the visible frequency range so as to exploit the solar’s energy efficiently. Parametric investigations with regard to the measurements of the design structure are fulfilled to characterize the absorber. The finite-difference time-domain (FDTD) method-based CST simulator was used to keep the pattern parameters and absorbance analysis. The metamaterial shows almost 99.96% and 99.60% perfect absorption at 523.84 THz and 674.12 THz resonance frequencies. Moreover, absorption’s FBW is studied, and 39.22% FBW is found. The results confirm that the designed PMA can attain very high absorption peak at two modes such as transverse electric (TE) and transverse magnetic (TM) mode. Other than the numerical outcomes demonstrated that the suggested configuration was also polarization angle insensitive. In addition, the change of absorbance of the structure has provided a new kind of sensor applications in these frequency ranges. Therefore, the suggested metamaterial absorber offers perfect absorption for visible frequency ranges and can be used for renewable solar energy harvesting applications.

## Introduction

Solar energy is a standout amongst other sustainable power source assets to create power with the assistance of photovoltaic (PV) cells. Power conveyed by the electromagnetic radiations inside one hour can meet yearly power utilization of the World [1]. In whatever case, however, the issue is that there is no adequate method to reap this asset. Metamaterial innovation can be employed to undertake the subject of electromagnetic energy harvesting by viably constructing more productive absorbers. These have on account of they have remarkable properties, for example, negative permeability, left-handed properties, negative refraction and evanescent wave amplification which cannot be found in natural materials [2,3], invisible cloaks [4], perfect lens [5], and a perfect absorption [6].

Because of the remarkable electromagnetic properties of various metamaterials, the design and use of metamaterial have picked up the need of fiery research [7]. However, Metamaterials are likewise being broadly considered for various applications, for example, perfect absorbers from mm to nm wavelengths [8], flexible metamaterials [9], multiband metamaterials [10] polarization insensitive absorber [11], broadband absorber [12], multiband absorber [13], and detectors [14], and Sensors [15]. There are numerous examinations that research PMAs. One case for these looks into being Tao et al. [16], in which they displayed the outline, manufacture, and characterization of a dual-band metamaterial absorber (MA). Their structure presented absorption peaks of 85% at 1.4 THz and 94% at 3.0 THz. Additionally, they presented [17] an MA that performs as a robust, resonant absorber at terahertz frequencies. Nevertheless, the experimental results of the absorption of their design structure are illustrated at 1.3 THz as 70%. Ahmed M. Montaser [18] proposed 1150 × 1150 nm^2^ with the thickness of 200 nm for visible frequencies. The high absorption of the structure was 99.46% and 99.4% at 265.8 THz and 556.4 THz, respectively. Dincer et al. suggested a PMA based on the square resonator with hole setup. It can be seen that the greatest absorptivity estimation of 99.92% is achieved at 0.865 THz [19]. On the other hand, they got 4.56% fractional bandwidth that shows the quality of suggested PMA. Hu et al. [20] numerically outlined a metamaterial that works in the THz band and having four narrowband high absorptivities of 98, 97, 98, and 97% at frequencies of 0.68, 1.27, 2.21, and 3.05 THz, separately. Ustunsoy et al. recommended a PMA unit cell structure with 500 × 500 nm^2^ dimensions which has 99.99% absorption at 558.75THz and 99% absorption at 216.75THz [2]. Li et al. offered concise and dual-band tunable perfect absorber based with a strontium titanate (STO) crystal substrate. The design structure exhibited a maximum absorption peak of 97.97% and 95.92% at 0.15 THz, and 0.30 THz, respectively [21]. Mulla et al. suggested a metamaterial-based absorber for solar energy harvesting. The absorption capability of absorber has 98.2% at 445.85 THz and 99.4% between 624 and 658.3 THz. Authors utilized aluminum (Al) as metallic parts of the structure and the silicon dioxide (SiO_2_) is a dielectric material [22].

In this paper, PMA displays double resonance at visible frequencies. The absorbance of the absorber is altered by varying the thickness of metallic materials and substrate, the polarization angle of design structure, resonator’s radius, and resonator’s shape with different and same effective area. Moreover, the fractional bandwidth of the suggested unit cell is 39.22%, that is more prominent than the expressed metamaterials structures in writing [2, 18]. Facilitate besides, and the outline structure gives high absorption peaks of 99.96% and 99.60% at 523.84 THz and 674.12 THz, separately. It is watched that the absorbance pinnacle of the suggested configuration is superior to the suggested metamaterials unit cells in [18, 21, 23]. The suggested design structure is very simple and flexible for rescaling that gives a chance to yield a multi-band absorber. The development of this design structure can efficiently guide the understanding of more effective photovoltaic solar cells.

## Structure, design and simulation setup of the PMA

**Fig 1:**
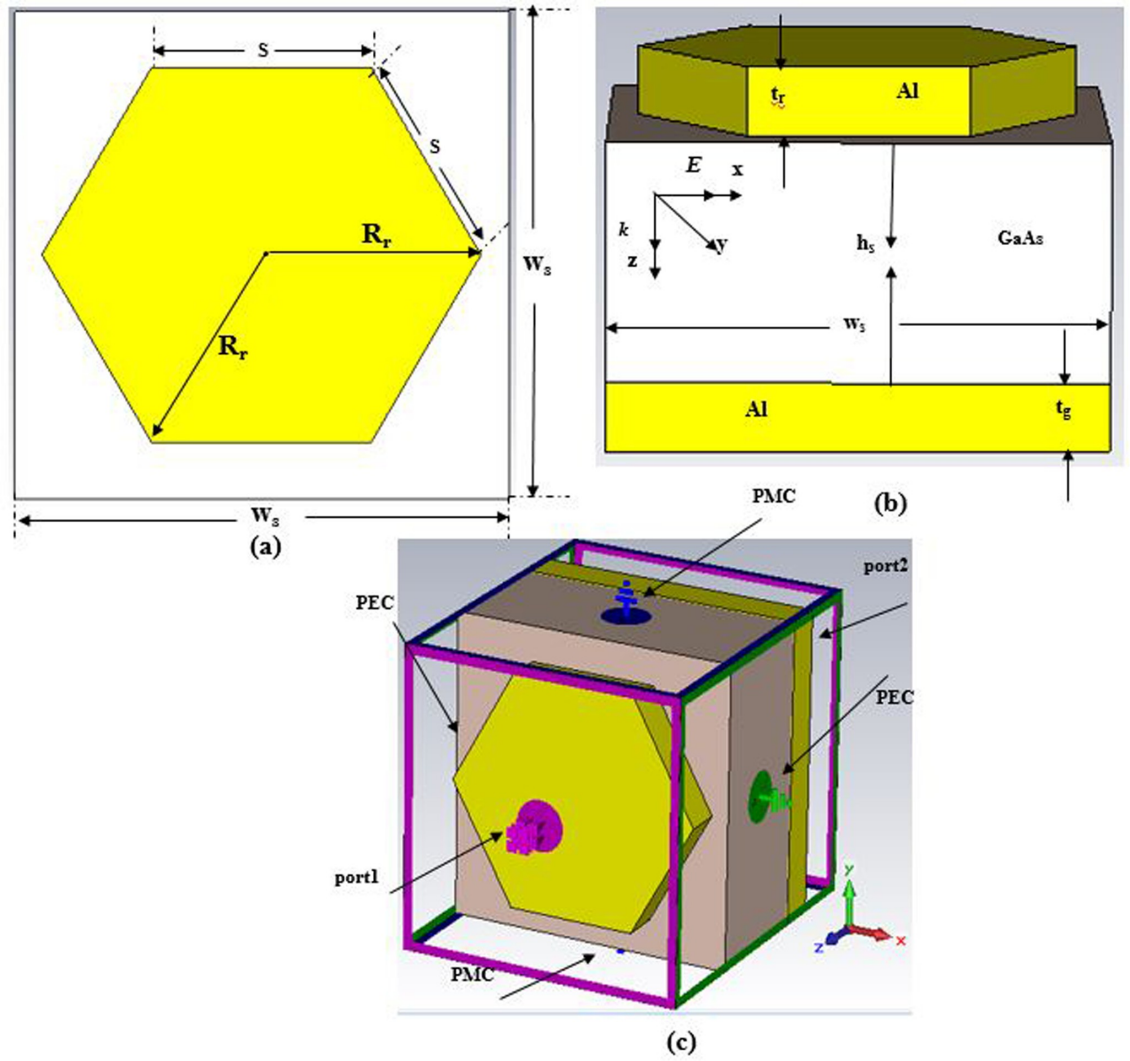
(a) Schematic diagram of PMA; (b) Unit cell with the bottom view; (c) Perspective view with boundaries.

The offered PMA is made out of two metal layers which are Al (Aluminum) separated by GaAs (Gallium Arsenide) layer as a dielectric substrate which is illustrated in Fig 1(A). Gallium arsenide (GaAs) is a vital semiconductor material that use to yield highly efficient solar cells. GaAs material has been utilized because of its various important properties such as high saturated electron velocity, high electron mobility, and a wide energy band gap which built it insensitive to temperature and heat. Also, GaAs has some advantages, namely, low-temperature coefficient, high-efficiency, good low light performance, excellent UV, radiation and moisture resistance, flexible and lightweight, etc. Besides, GaAs has a direct band gap that makes it a good solar energy absorber. The specifications of Al are σ_aluminum_ = 3.56×10^7^ S/m, Rho = 2700 [kg/m^3^], thermal conductivity = 237 [W/K/m], and GaAs’s specifications are ε_GaAs_ = 12.94, tanδ = 0.006, Rho = 5320 [kg/m^3^], and thermal conductivity = 54 [W/K/m]. The schematic geometry with the bottom view of the structure is depicted in Fig 1(B).

The uppermost hexagonal disk nanoantennas are periodically spread with a resonator of 566 nm along the x- and y-axis. The other parameters of the hexagonal nanoantenna are t_r_ = t_g_ = 75 nm, h_s_ = 260 nm, w_s_ = l_s_ = 566 nm and R_r_ = 250 nm that are shown in Table 1. Background thickness of aluminium layer is noteworthy since it works as a photosensitive reflector to near zero transmittance. This thickness of the background Al layer additionally couples with resonator-top Al layer that is exhibited in Fig 1(B) to create dipole moment. Fig 1(C) indicates the design structure with boundary conditions.

**Table 1:** Parameters of the suggested PMA structure.

Parameters	Value(nm)	Parameters	Value(nm)
Substrate width, w_s_	566	Resonator’s sidearm, s	250
Substrate length, l_s_	566	Resonator’s thickness, t_r_	75
Substrate height, h_s_	260	Ground plane’s thickness, t_g_	75
Resonator’s radius, R_r_	250		

## Fundamental theory of the PMA

Absorbance characteristics of PMA structure depicted by two fundamental parameters, such as, reflectance (R (ω)) and transmittance (T (ω)) which are frequency related quantities. The expression [24] of the PMA is ***A***(***ω***) = **1** − ***R***(***ω***) − ***T***(***ω***).

The rate of the reflectance and transmittance of the absorber depends on the scattering parameters, ***R***(***ω***) = ***S***_**11**_^**2**^ and ***T***(***ω***) = ***S***_**21**_^**2**^, which are mentioned at bellow,

$$S_{11}=\frac{\sqrt{Reflected\;power}}{\sqrt{Incident\;power}};\;S_{21}=\frac{\sqrt{Transmitted\;power}}{\sqrt{Incident\;power}}$$

To adjust the absorbance of PMA structure, the coefficients of reflection and transmission have to minimize to get minimum viable values. In order to this process, the most common technique is to engage in a combination of the periodic arrangement of the resonator on top of the dielectric and metal-dielectric layers. Therefore, optimization of PMA geometric structure, at the resonance frequency where ***Z***(***ω***) = ***Z***_**0**_, at this point, the impedance can be matched with free space impedance.

## Parasitic analysis, results, and discussion of the PMA

**Fig 2:**
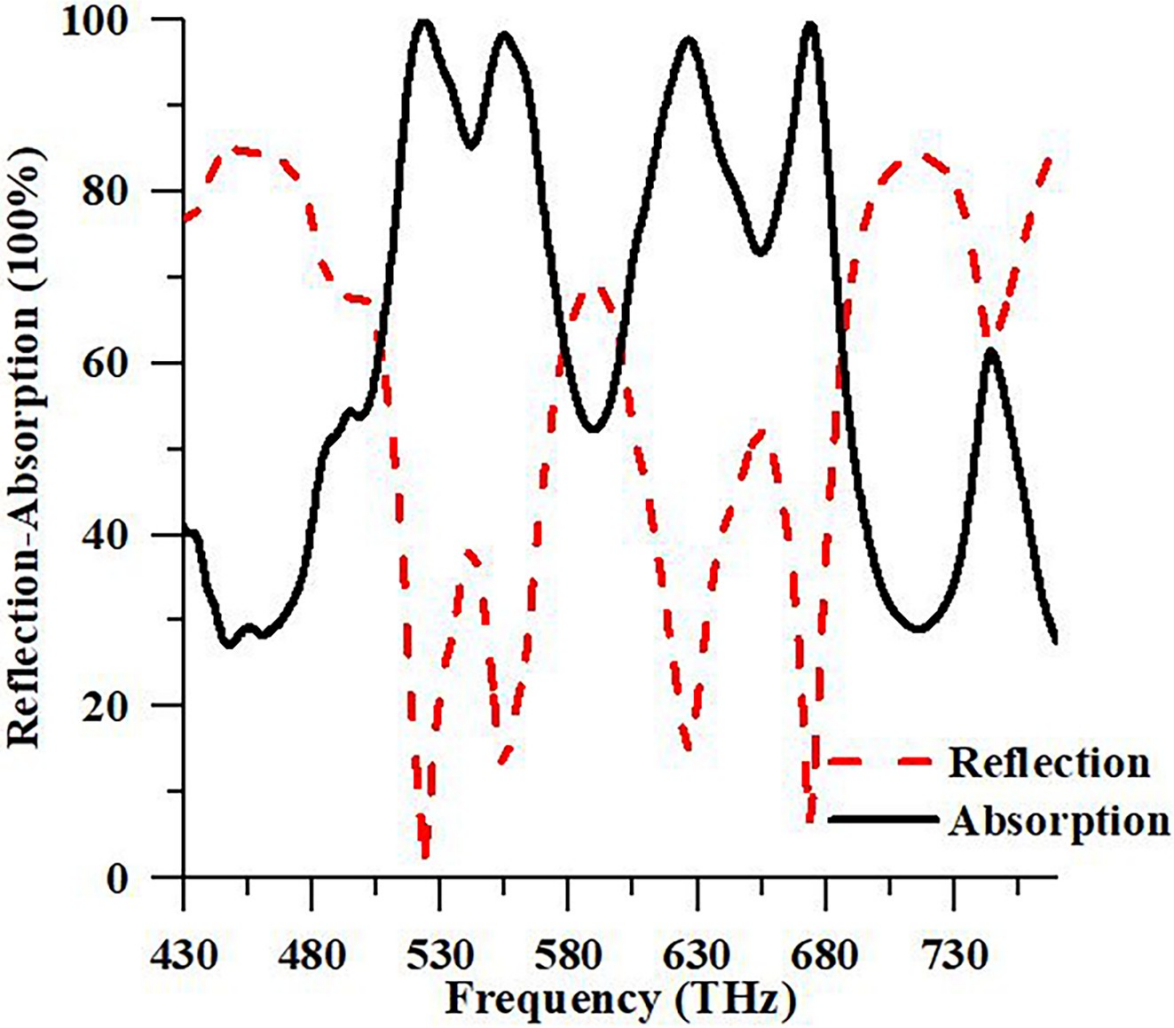
Numerical reflection and absorbance spectrum of suggested Structure.

Numerical is observed using the FDTD method-based CST EM simulator 2015 to inspect the structure in this study. A unit cell boundary condition is considered along x and y directions while an open boundary is in the z-direction in the boundary condition setup. The propagation direction of the electromagnetic wave is along the z-axis with the electric and the magnetic field parallel to the x- and y-axis, respectively. The diagram’s illustration of the suggested structure, structure with front view, the structure with boundary condition is exhibited in Fig 1. For analysis, the performance of the structure and array of structure in the numerical simulations, frequency domain solver is utilized. In this manuscript, reflectance and the absorbance parameters, the absorbance of design structure by changing the thickness of Al metals and GaAs, the effect of the resonator’s shape, resonator’s radius, the effect of polarization based on TE and TM have been observed. The numerical absorbance and reflectance of the suggested structure are shown in Fig 2.

It is observed in Fig 2, the range of frequencies of the suggested structure is in the visible regime. On the other hand, other recommended design structures in literature have small FBW and less absorbance, and the suggested PMA has high FBW, compact, high peak, full width at half-maximum (FWHM), and perfect absorptivity. Moreover, Fig 2 exhibits the numerical absorbance spectrum of the suggested PMA. From Fig 2, it can be seen that the maximum absorbance peaks are 99.96% and 99.60%, which are located at 523.84 THz and 674.12 THz. The absorbance bandwidths of PMA are FWHM that is 63.58 THz and 79.56 THz, respectively, which is higher than Li et al. authors’ paper [21].

**Fig 3:**
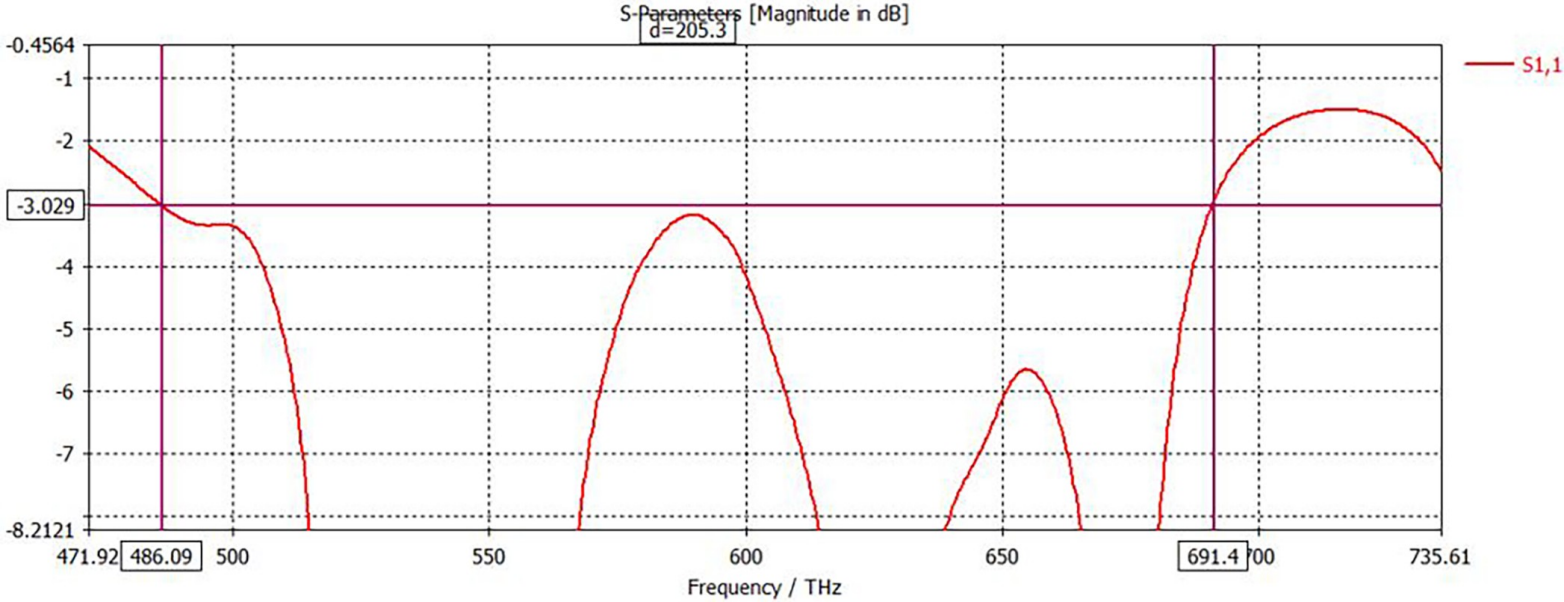
Fractional bandwidth curve of the proposed PMA.

From Fig 3, the FBW of the suggested absorber is calculated. FBW can be determined as Δff0, where the half-power bandwidth and center frequency are Δ*f* and *f*_0_. The quality of the absorber can be determined by using FBW data [19]. It can be seen from Fig 3, bandwidth, Δ*f* = 205.30 THz, first central frequency, *f*_0_ = 523.84 THz, and FBW ≈ 39.22% for the first resonance. For the second resonance, Δ*f* = 205.30 THz, *f*_0_ = 573.78 THz, and FBW ≈ 30.47%. It can be observed from the results, FBW comparatively very high both of resonance frequency to the mentioned paper in this literature.

**Fig 4:**
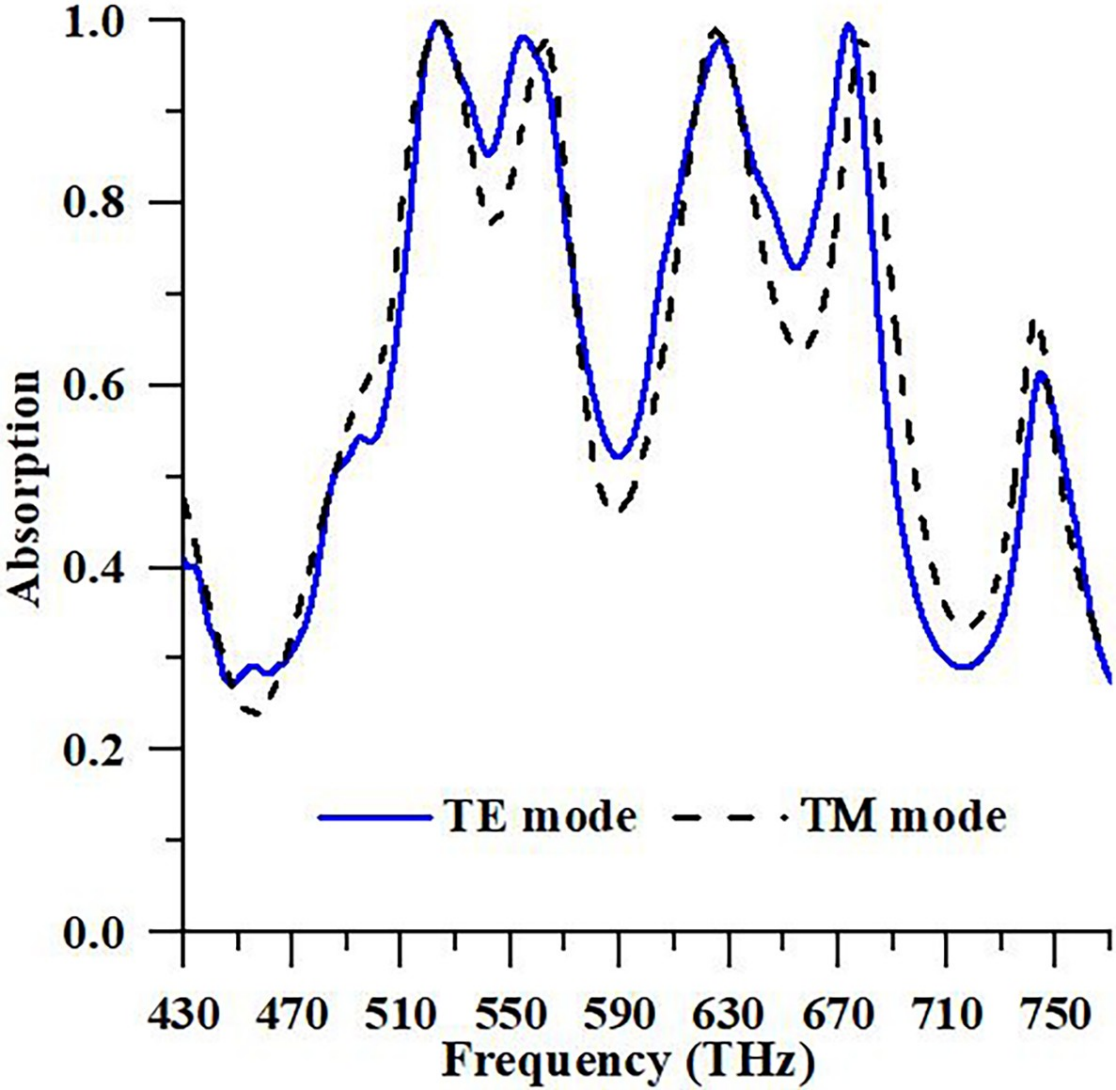
Numerical response of the proposed PMA in case of TE and TM polarization.

**Fig 5:**
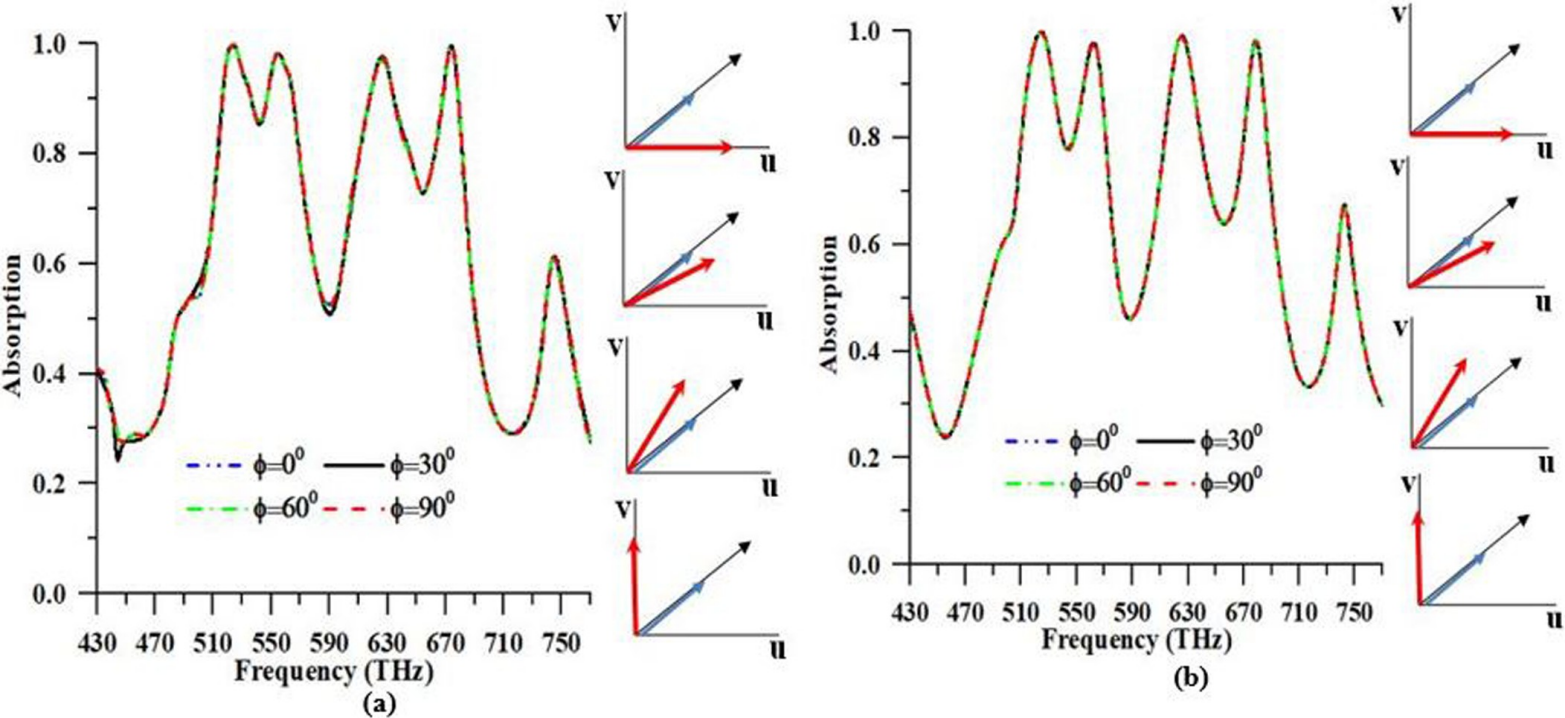
**Numerical response of PMA for (a) TE and (b) TM polarization**.

In numerical analysis, the observation of solar energy polarized in Transverse Magnetic (TM) and Transverse Electric (TE) is accomplished using periodic boundary condition. In Fig 4, absorbance characteristics of the suggested PMA for TM and TE mode polarizations are illustrated. It is seen from the Fig 4 that the suggested PMA is autonomous for TM and TE polarization due to the symmetrical design structure. The absorbance peaks are 99.95% and 99.12% at resonance frequency 524.18 THz and 625.5 THz, respectively in TM mode. On the other hand, Maximum peaks of absorbance are 99.96% and 99.60% at resonance frequency 523.84 THz and 674.12 THz, individually in TE mode. Besides, the solar absorber based on PMA structure is polarization and frequency individualistic with an absorption rate over 80% between 430 and 770 THz. To realize the absorbent properties of the design, identifying to the supply of EM wave, the characteristic is understood at different polarization angles of EM ray that are revealed in Fig 5.

In the study of the PMA structure for photovoltaic solar cell applications, the fulfillment of design structure over the different polarization of electromagnetic (EM) solar radiation plays a serious role. To make a solar cell for capturing more solar energy has remained a challenge to contain EM radiation using arbitrarily polarized apparatuses. The absorption rate under different polarizations such as TE and TM mode are shown in Fig 5, where the numerical analyses were accomplished under different angles varying from 0° to 90° at a 30° interval. To analysis, the incident radiation of TE mode, EM radiation incident angles are being altered whereas EM radiations electric component are continuously kept tangential to the surface of the PMA structure. Similarly, for incident radiations of TM mode, EM radiations magnetic component steadily fixed tangential to the surface of PMA structure for all EM radiation incident angles. In numerical analysis, one can see that the maximum absorbance peaks are 99.96%, 99.97%, 99.95%, and 99.95% at 523.84 THz for different polarization angle likes 0^0^, 30^0^, 60^0^ and 90^0^ respectively, in case of TE mode. Moreover, the maximum peaks of absorbance are 99.86%, 99.86%, 99.86%, and 99.86% at 523.84 THz for different polarization angle like 0^0^, 30^0^, 60^0^ and 90^0^ respectively, in case of TM mode that is shown in Table 2. It is also seen in Fig 5(A) and 5(B), the suggested design structure provides near fixed and perfect absorbance in order to all angles of the incident electromagnetic wave. In Fig 5(A) and 5(B), the absorption reply of the suggested structure depicts that suggested structure angle individualistic the two instances of TE and TM waves. The absorbance of suggested PMA is over 70% in the visible spectra with polarization adaptability. Hence, the suggested PMA is a decent contender for the comprehension of dynamic absorber gadgets that are working in unmistakable daylight frequency spectra and can likewise be employed as a vitality collector for sun-oriented daylight applications because of inhumanity for TE and TM energized waves.

**Table 2:** Comparison between the TE and TM polarization mode in terms of max^m^ absorption peaks.

Polarization mode	Polarization angle (deg)	Max^m^ absorption peaks
**TE mode**	0^0^	99.96% at 523.84 THz
	30^0^	99.97% at 523.84 THz
	60^0^	99.95% at 523.84 THz
	90^0^	99.95% at 523.84 THz
**TM mode**	0^0^	99.86% at 523.84 THz
	30^0^	99.86% at 523.84 THz
	60^0^	99.86% at 523.84 THz
	90^0^	99.86% at 523.84 THz

**Fig 6:**
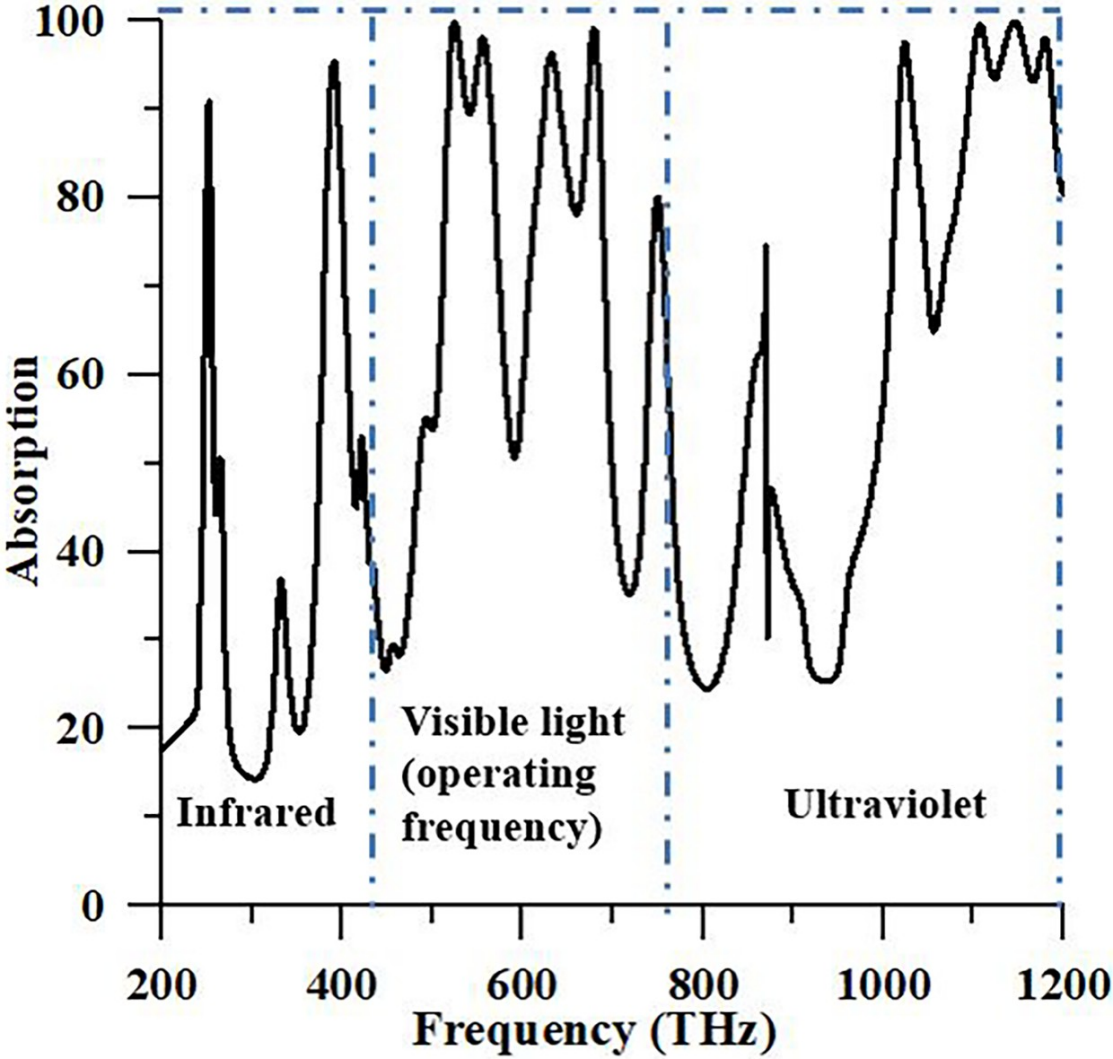
Absorption reply of the suggested PMA for whole sunlight.

The absorbance characteristic of the suggested PMA structure examined for all sunlight such as infrared (200–430 THz), visible (430–770 THz) and ultraviolet (770–1200) frequency spectra. Fig 6 depicts the absorbance characteristic of suggested structures for entire sunlight spectra (200–1200 THz) is shown. It is seen from Fig 6, the different absorbance peaks available in infrared, visible and ultraviolet regions. It is seen from Fig 6 that absorbance over 70% between 380.50 THz and 402.5 THz for the infrared region; 511.4 THz– 575.2 THz and 607.60 THz– 691.8 THz for visible frequency region; 1008.60 THz– 1049.3 THz and 1065.8 THz– 1200 THz for the ultraviolet region. From Fig 6, it can be observed in numerically that suggested configuration structure portrays idealize absorption properties among infrared, visible and ultraviolet light spectrum. This feature of suggested PMA likewise makes special it from another structure in writing as far as sun-based vitality application as pinnacle absorber.

In this portion, a parametric study is observed. The reliance of absorbance on the geometric parameters of the suggested PMA is investigated as detail. In order to perceive the response of the PMA by altering of different thickness of the top and bottom metal (Tmetal), and radius of top metal (Rr) inside sensible breaking point to realize the instrument of PMA, however, different parameters are retained unaltered of PMA. This characteristic offers machine-driven adjustable attribute of the PMA. To acquire the ideal estimation for suggested design, top and bottom metal thickness, and radius of the top are changed and the numerical analysis conducted, simultaneously.

**Fig 7:**
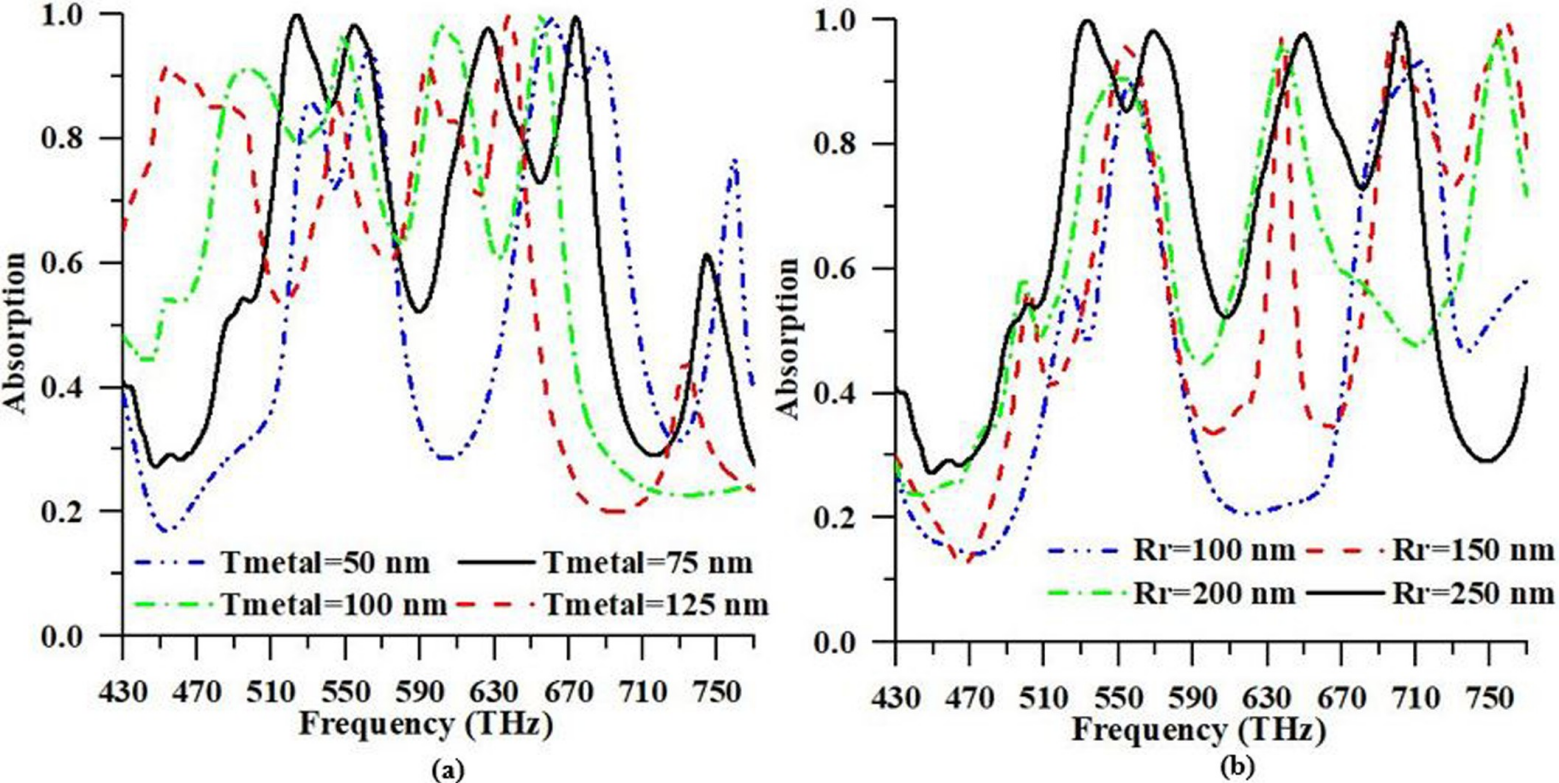
**Absorption response of the proposed PMA for different (a) resonator and ground metal thickness (from 50 nm to 125 nm) and (b) radius of the resonator (from 100 nm to 250 nm)**.

**Fig 8:**
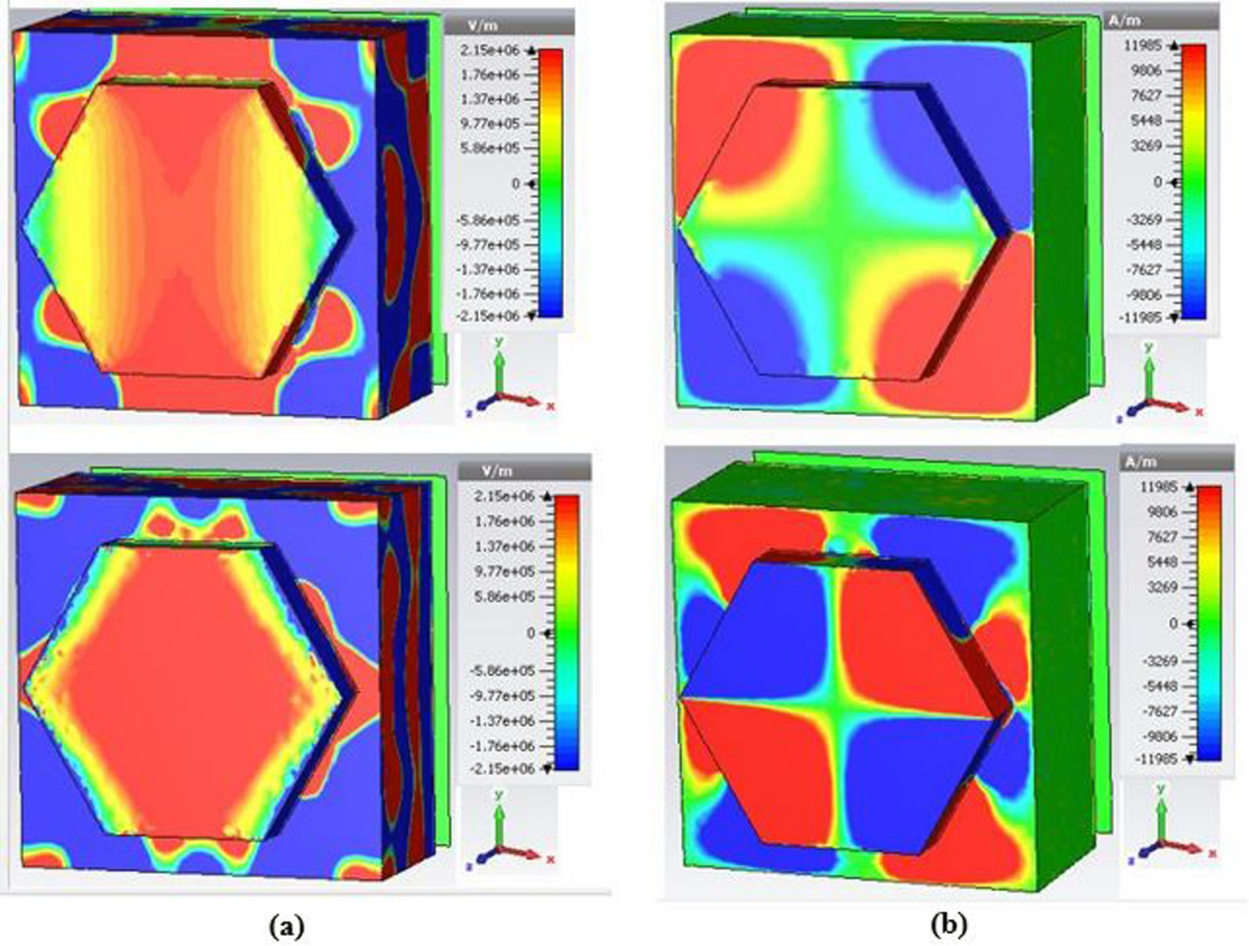
(a) Electric field; (b) Magnetic field distributions of PMA at resonance frequency 523.84 THz and 674.12 THz, respectively.

Fig 7(A) and 7(B) demonstrates the absorptivity properties at the different amplitude of top and bottom metal thickness and radius of top metal of the design structure, respectively. Color curves in Fig 7(A) and 7(B) display the desired outcome of absorbance. In this manner, these outcomes' parameters can be picked as ideal parameters for the suggested PMA to idealize absorbance. It is seen in Fig 7(A), the high absorbance peaks (99.96% and 99.60%) are obtained at resonance frequencies for the value (75 nm) of the top and bottom metal thickness. On the other hand, other thickness of top and bottom metal shows the lower absorbance peaks. The radii from r = 100 nm to r = 250 nm of the resonator is varied where a small enhancement in absorbance rate is investigated for large values of resonator radius. This is for, the absorbance capability of the suggested design structure relies on the coupling between a resonator size and its surrounded substrate material. Hence, at certain values of Rr, strong coupling between the resonator and its embedded substrate material causes a strong electromagnetic field which leads to alter the absorbance rate at the resonant frequencies. In addition, from Fig 7(B), it is observed that the high absorbance peaks are obtained at resonance frequencies for the value (250 nm) of the resonator’s radius. In this portion, the distributed electric and magnetic field of the suggested PMA is examined at the resonance frequencies of 523.84 THz and 674.12 THz, respectively. It will assist to realize the operation principle of the suggested PMA structure. To understand the physics behind the absorbance characteristics at the resonance frequency, the electric field and the magnetic field distributions of the suggested PMA structure are observed. Electric field distributions for two main resonant frequencies (523.84 THz and 674.12 THz) can be found in Fig 8(A).

In Fig 8(A) the strong electric fields are allocated in the upper and lower sides of the top metal resonator layer. It is also seen that the E-field arrangements like symmetric shape upper and lower sides of the resonator for the first resonant frequency of 523.84 THz, which performs as an electrical dipole. It is also observed in the second resonance frequency (674.12 THz), the E-field distribution concentrated at the center of the resonator and right side in the embedded substrate material which also performs an electrical dipole. Hence, the coupling of the electric field between the resonator and ground plane makes the autonomous electric resonance. Because of electric resonance, the electric field at 523.84 THz is stronger than at 674.12 THz resonance frequency. Those electrical dipoles are responsible for getting more absorption. Moreover, magnetic field distributions for two main resonant frequencies (523.84 THz and 674.12 THz) can be realized in Fig 8(B). It is also recognized that the M-field arrangements like symmetric configuration on the corner of the resonator and embedded substrate material for the first resonant frequency of 523.84 THz, which acts as a magnetic quadrupole. It is also seen in the second resonance frequency of 674.12 THz, the M-field distribution concentrated in the corner of the resonator and embedded substrate material which also works a magnetic octupole. Hence, the coupling of the magnetic field between the resonator and ground plane makes the self-directed magnetic resonance. This magnetic resonance is caused to obtain more absorption at 523.84 THz compare to at 674.12 THz.

**Fig 9:**
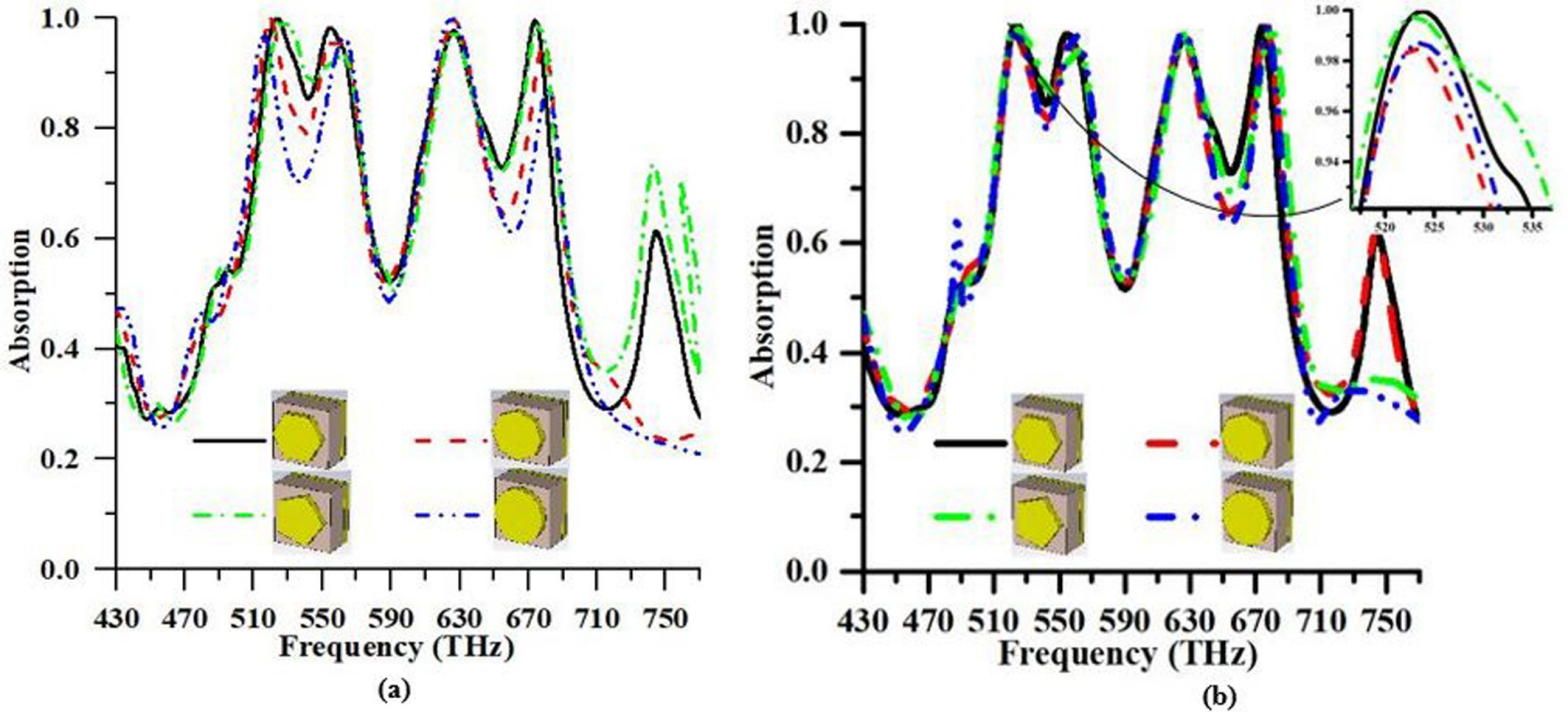
**Absorption characteristic of the proposed PMA for different resonator shape such as hexagonal, octagonal, pentagonal, and circular with (a) the different effective area (b) the same effective area**.

Keeping in mind the end goal to watch the response of different patch shape of the resonators like hexagonal, octagonal, pentagonal and the circular shape with the different effective area, but same resonator radius is created and analyzed individually. Fig 9(A) delineates the impacts are contrasted and examined with the suggested structure. The most extreme absorbance peaks are 99.96%, 99.90%, 99.50%, and 96.83% in order to first resonance at 523.84 THz, 519.76 THz, 526.9 THz, and 516.36 THz respectively for different shape such as hexagonal, octagonal, pentagonal, and circular. On the other hand, 99.58%, 99.71%, 98.83%, and 99.91% are the high peak absorption of the second resonance at 674.12 THz, 626.86 THz, 675.82 THz, and 625.50 THz, simultaneously. Fig 9 depicts that hexagonal shape is more suitable to apply for solar energy harvesting in terms of high absorption peaks and more FBW.

In addition, the response of different patch shape of the resonators like hexagonal, octagonal, pentagonal and the circular shape with the same effective area (162,379.76 nm), but different resonators radius are made and analyzed independently. Fig 9(B) also depicts the impacts are compared and observed with the suggested structure. The most extreme absorbance peaks are 99.96%, 98.48%, 99.76%, and 98.70% in order to first resonance at 523.84 THz, 523.16 THz, 523.16 THz, and 523.50 THz respectively for different shape such as hexagonal, octagonal, pentagonal, and circular. In contrast, 99.60%, 99.98%, 99.62%, and 99.37% are the high peak absorption of the second resonance at 674.12 THz, 676.50 THz, 679.56 THz, and 676.84 THz, simultaneously. Fig 9 shows that hexagonal shape is more appropriate to gain solar energy because of high absorption peaks, more FBW, and more FWHM bandwidth. The detail comparison among the shapes is shown in Table 3.

**Table 3:** Comparison of the different shapes in terms of the effective area.

Category	Shape of the structure	Effective Area (nm^2^)	Radius of the resonator (nm)	Max^m^ absorption peaks at resonance frequencies
**Different effective area nonetheless same resonator’s radius**	Hexagonal	162379.76	250.00	99.96% and 99.60% at 523.84 THz and 674.12 THz, respectively
	Octagonal	176776.69	250.00	99.90% and 99.71% at 519.76 THz and 626.84 THz, respectively
	Pentagonal	148620.58	250.00	99.50% and 98.83% at 526.90 THz and 685.82 THz, respectively
	Circular	196349.54	250.00	96.83% and 99.91% at 516.36 THz and 625.50 THz, respectively
**Same effective area nonetheless different resonator’s radius**	Hexagonal	162379.76	250.00	99.96% and 99.60% at 523.84 THz and 674.12 THz, respectively
	Octagonal	162379.76	239.60	98.48% and 99.98% at 523.16 THz and 676.50 THz, respectively
	Pentagonal	162379.76	261.332	99.76% and 99.62% at 523.16 THz and 679.56 THz, respectively
	Circular	162379.76	227.358	98.70% and 99.37% at 523.50 THz and 676.84 THz, respectively

**Fig 10:**
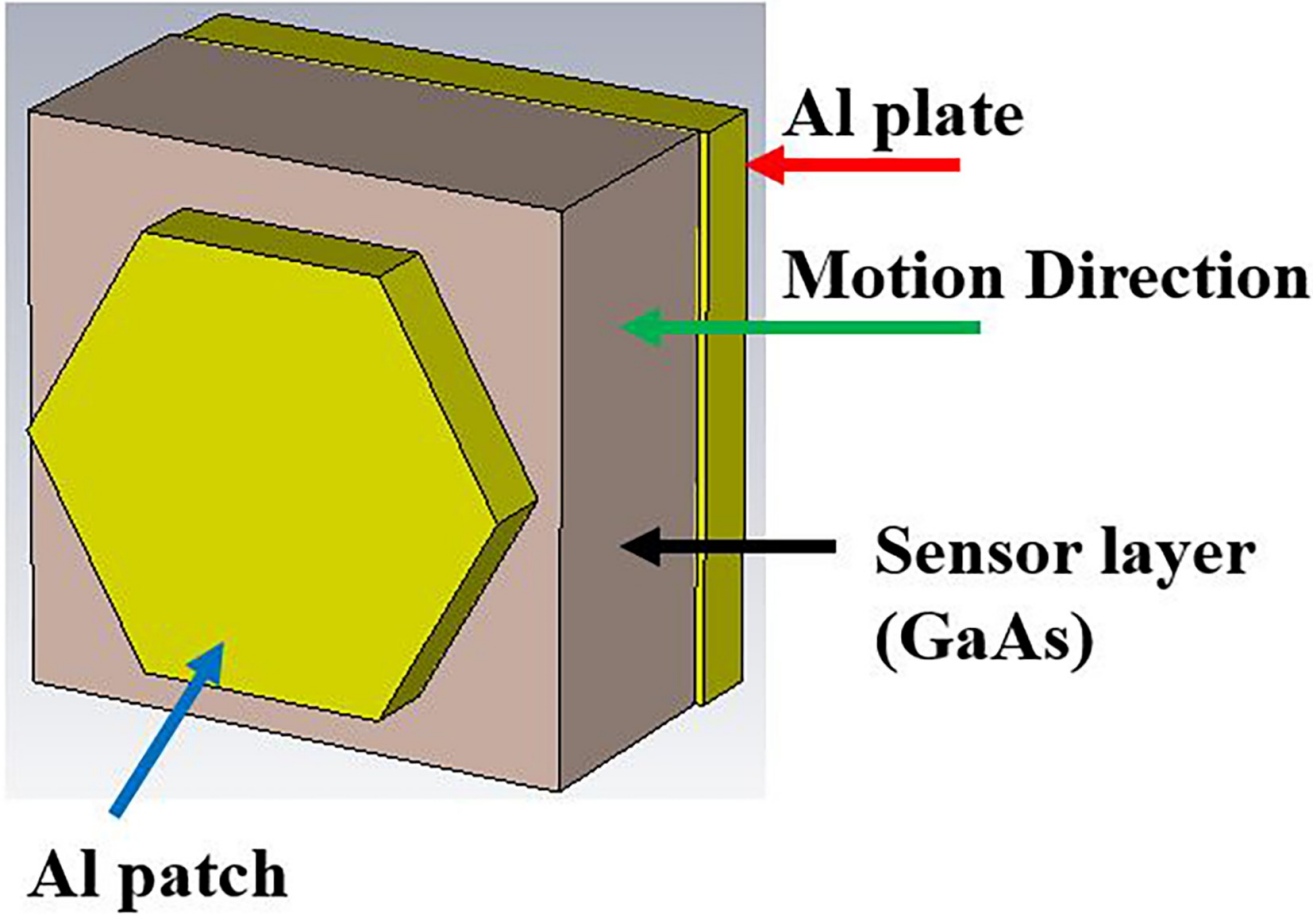
Sensor application block diagram.

**Fig 11:**
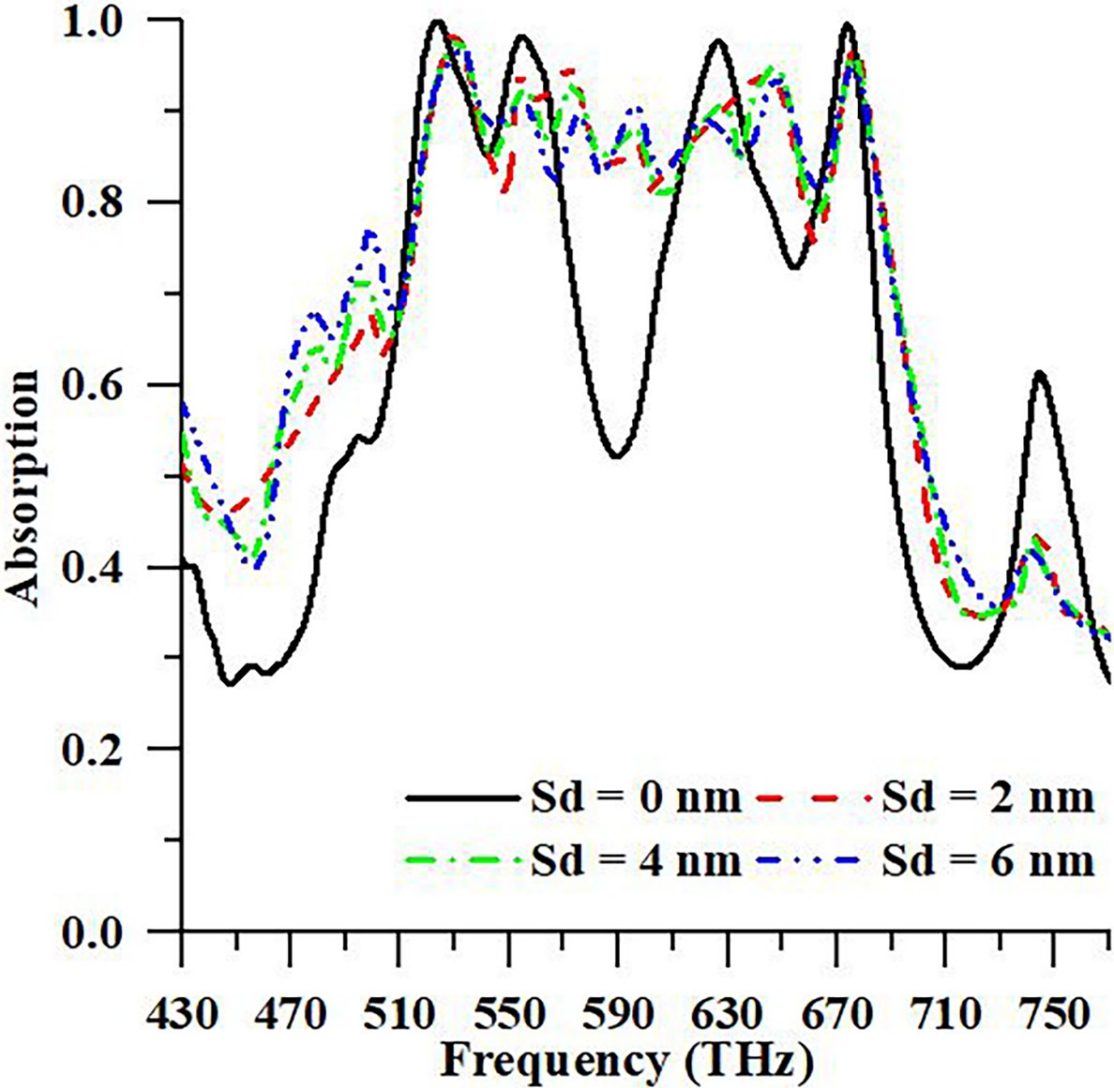
Absorption characteristic of the proposed PMA for different displacements like 0 nm, 2 nm, 4 nm, and 6nm between conducting materials.

In this portion of the study concentrates on a sensor application of PMA. In order to understand a sensor application, a block schema is shown in Fig 10. The sensor has to encounter some necessary requirements to work efficiently. Firstly, the sensor has to offer a measurable signal according to variations in the parameters. Secondly, linearity that is indicated the response of the sensors must be linear according to value changes in the observed parameters. The last one is sensor sensitivity that means change the parameters of the design structure can be observed easily by the network analyzer Sensing structure is placed between the resonator and the ground plate in the suggested design sensor structure. The sensing structure is modeled as a material having variable material parameters. Meanwhile, any small change like displacement, thickness, etc. effects of resonance character. In this study, the design structure acts as a displacement sensor. The material changes the position between resonator and ground plate from 0 nm to 6 nm by using the 2 nm step. It is observed in Fig 11 that the maximum absorption peaks are 99.96%, 93.42%, 93.48%, and 93.15% at 523.84 THz resonance frequency in order to the displacement of GaAs material such as 0 nm, 2 nm, 4 nm, and 6 nm, respectively.

The contrasts with suggested PMA and another offered PMA are depicted in Table 4. The thought about the parameters of PMA have been well-thought-out here, for instance, Number of absorption peak, the dimension of the structure, cover frequency range, FBW, absorbance percentage and year of published. Mulla et al. [2], offered brick-shape structure with 420 × 155 nm^2^ dimensions and attained FBW by 22.2% at 403.5 THz in Table 1. Nevertheless, other authors, Montaser et al. in [17], recommended hexagonal shape absorber for all bands from microwave to terahertz application. On the other hand, the author has attained 99.4% at 556.4 THz absorptions with 1150 × 1150 nm^2^ sizes of design structure and FBW of 4.64% at 2.8 GHz. Li et al. [20] revealed a square shape with 40 × 6 μm^2^ dimensions of configuration structure which has achieved an absorbance peak of 97.97% at 0.15 THz and 95.92% at 0.30 THz. However, the authors have not shown FBW in their literature. In [3] Square-shape metamaterial absorber analyzed by Ustunsoy et al. and have been gained a small amount of FBW like 20.74% at 141 THz. Conversely, authors have acquired the highest peak of absorption such as 99.99% at 568.75 THz and 99% at 216.75 THz. Bağmancı et al. [22] suggested cross shape structure with 500 × 500 nm^2^ sizes for 430–770 THz frequency range and acquired absorbance of 99% at 216.75 THz. The miniaturize hexagonal shape PMA has been investigated and obtained peak absorbance (99.96% at 523.84 THz and 99.60% at 674.12 THz) with highest FBW (39.22% at 523.84 THz) in this paper. The suggested PMA has gained high absorption and a large amount of FBW comparing stated references that are suitable for the visible regime.

**Table 4:** Comparison between the previous PMAs and suggested PMA.

Author name	Mulla et al. [2]	Ustunsoy et al. [3]	Montaser [18]	Li et al. [21]	Bağmancı et al. [23]	Hossain et al.
**Design structure**	Brick shape	Square shape	Hexagonal shape	Square shape	Cross shape	Hexagonal shape
**Number of the absorption peak**	1	2	3	2	1	2
			1			
			1			
**Dimension**	420 × 155 nm^2^	500 × 500 nm^2^	30 × 30 mm^2^	40 × 6 μm^2^	500 × 500 nm^2^	566 × 566 nm^2^
			2750 × 2750 nm^2^			
			1150 × 1150 nm^2^			
**Cover band**	350–455 THz	0–600 THz	2.5–6 GHz	0.1–0.5 THz	430–770 THz	430–770 THz
			264–268 THz			
			255.4–257.4 THz			
**FBW**	22.2% at 403.5 THz	20.74% at 141 THz	4.64% at 2.8 GHz	—	—	39.22% at 523.84 THz
			—			
			—			
**Absorption rate**	99.99% at 403.5 THz	99.99% at 568.75 THz	99% at 2.8 GHz	97.97% at 0.15 THz	99% at 216.75 THz	99.96% at 523.84 THz
			99.46% at 265.8 THz			
		99% at 216.75 THz	99.4% at 556.4 THz	95.92% at 0.30 THz		99.60% at 674.12 THz
**Published Year**	2015	2016	2016	2016	2017	—

## Conclusions

A high FBW hexagonal PMA was offered to investigate the solar energy absorbance and sensing characteristics with the numerical outcomes. The offered structure was extremely basic, and it displayed high absorbance (99.96%) with high FBW (39.22%) in the visible frequency ranges. The offered configuration has shown high absorption of infrared, visible light and ultraviolet part. Thus, the essential and adequate circumstances for polarization- autonomous behavior of PMA having high absorptivity can be advocated by impedance matching. It is observed that the suggested PMA is unresponsive to the angle of incidence for polarization mode of TE and TM amid the entire of the working frequency spectra. The properties of the suggested PMA are incredibly significant meanwhile the suggested model has numerous focal points contrasted with those in writing. In addition, the distributions of the electric and magnetic field are presented and investigating to realize better and exhibit the absorbance phenomena. A similar examination likewise went up against the premise of the number of absorption peak, dimension, covers band, FBW, absorption rate and published Year. The specified variable of PMA demonstrates excellent execution and the estimations of absorbance are round unity. Besides, the suggested PMA will lead to the understanding of high-efficiency solar cells and other gadgets operating in the high-frequency spectral regime with polarization angle insensitive. The metamaterial structure was miniaturized in dimension, and unity absorbance, which gives it perfect for solar energy harvesting, sensors, and wireless communications.
