# Correction: Perfect metamaterial absorber with high fractional bandwidth for solar energy harvesting

**DOI:** 10.1371/journal.pone.0211751

**Published:** 2019-01-29

**Authors:** Mohammad Jakir Hossain, Mohammad Rashed Iqbal Faruque, Mohammad Tariqul Islam

There are errors in the Funding section. The correct funding information is as follows: This study was funded by the Universiti Kebangsaan Malaysia grant DPP-2018-134.The funders had no role in study design, data collection, and analysis, decision to publish, or preparation of the manuscript.

## References

[1] Hossain MJ, Faruque MRI, Islam MT (2018) Perfect metamaterial absorber with high fractional bandwidth for solar energy harvesting. PLoS ONE 13(11): e0207314 10.1371/journal.pone.0207314 30419057 PMC6231668

